# Impact of the Anatomical Accelerometer Placement on Vertical Jump Performance Characteristics

**DOI:** 10.3390/sports11040092

**Published:** 2023-04-21

**Authors:** Damjana V. Cabarkapa, Dimitrije Cabarkapa, Nicolas M. Philipp, Andrew C. Fry

**Affiliations:** Jayhawk Athletic Performance Laboratory—Wu Tsai Human Performance Alliance, Department of Health, Sport and Exercise Sciences, University of Kansas, Lawrence, KS 66045, USA

**Keywords:** sport, wearable technology, testing, assessment, performance, force

## Abstract

With rapid technological development over recent years, the use of wearable athlete monitoring devices has substantially gained popularity. Thus, the purpose of the present study was to examine the impact of the anatomical placement of an accelerometer on biomechanical characteristics of countermovement vertical jump with and without an arm swing when compared to the force plate as a criterion measure. Seventeen recreationally active individuals (ten males and seven females) volunteered to participate in the present study. Four identical accelerometers sampling at 100 Hz were placed at the following anatomical locations: upper-back (UB), chest (CH), abdomen (AB), and hip (HP). While standing on a uni-axial force plate system sampling at 1000 Hz, each participant completed three non-sequential maximal countermovement vertical jumps with and without an arm swing. All devices recorded the data simultaneously. The following variables of interest were obtained from ground reaction force curves: peak concentric force (PCF), peak landing force (PLF), and vertical jump height (VJH). The findings of the present study reveal that the most appropriate anatomical locations to place the accelerometer device when attempting to estimate PCF, PLF, and VJH during a countermovement vertical jump with no arm swing are CH, AB, and UB, and during a countermovement vertical jump with an arm swing are UB, HP, and UB, respectively. Overall, these findings may help strength and conditioning professionals and sports scientists to select appropriate anatomical locations when using innovative accelerometer technology to monitor vertical jump performance characteristics.

## 1. Introduction

The vertical jump is one of the most commonly used methods for the assessment of an athlete’s lower-body muscular power [1,2]. Given that numerous team sports (e.g., volleyball, basketball, soccer, American football) contain a vertical jump component, many strength and conditioning professionals and sports scientists consider it to be one of the essential factors related to successful athletic performance [3,4,5,6,7].

Over the years, various technologies (e.g., force plates, motion capture systems, accelerometers) have been used for the assessment of vertical jump performance characteristics (e.g., jump height, peak concentric force, impulse). Laboratory-based force plate technology has been considered the criterion measure or “gold standard” testing modality for in-depth analysis of vertical jump performance characteristics [8,9]. However, despite being highly reliable, this technology appears to be unsuitable for field assessments due to its low portability, high costs, and requirement of specialized computer equipment for data collection and analysis. Thus, to help strength and conditioning professionals and sports scientists efficiently administer vertical jump assessments on the field, different portable devices such as contact mats, optical timing systems, and accelerometers have often been implemented [10,11,12,13,14].

A considerable amount of scientific literature has examined the validity and reliability of various accelerometer-based technologies for the assessment of vertical jump performance characteristics and has reported mixed findings [10,11,13,14,15,16,17,18,19]. In a recently published study, Cabarkapa et al. [16] found that an innovative accelerometer device (StriveTech) was an acceptable testing modality for the assessment of vertical jump height, despite the tendency to overestimate measurements by approximately 3.1 cm when compared to the laboratory-based force plate system as a criterion measure. Similarly, the Myotest accelerometer was shown to overestimate vertical jump height by 8.0 cm and flight time by 6.4% when compared to the force plate [11,19]. Conversely, Rago et al. [13] indicated that the same device underestimated vertical jump height measurements when compared to a marker-based motion capture system. Moreover, the authors noted that its use is appropriate when measuring contraction time and eccentric duration, while optical measuring technology (Optojump) should be the preferred testing modality when assessing vertical jump height and flight time [13]. On the other hand, Hojka et al. [19] suggested that the accelerometers should not be used for measuring countermovement vertical jump kinetic characteristics due to a significant systemic bias toward underestimating peak force, power, and velocity by 167 N, 843 W, and 0.56 m·s^−1^, respectively [19]. However, when fixed to the barbell, this same device was shown to be a valid and reliable tool for measuring force and power production during a barbell back squat and bench press exercise [18].

Another important factor that needs to be considered when using accelerometer technology is its anatomical placement. Depending on the different manufacturers and their recommendations, the accelerometer can be placed on various body locations (e.g., the scapulae, abdomen, sternum, hip, thigh). Previous research reports have suggested that if the goal is to test performance on sport-specific skills, the accelerometer should be attached to the body segment that is performing a specific movement [20,21,22,23]. On the other hand, if the goal is to track a player’s position, orientation, velocity, and acceleration on the court/field throughout a practice session and/or game, the accelerometer should be placed on the scapulae [20,21,22,23]. However, other anatomical placements (e.g., sternum, wrist, head, abdomen, hip) have been used to examine athletes’ performance on sport-specific skills as well as quantify the overall workload [22]. For example, in a recently published study, Cabarkapa et al. [24] examined differences between the abdomen (i.e., 5 cm inferior to the umbilicus) and hip (i.e., 5 cm above the greater trochanter) accelerometer placements for the assessment of vertical jump biomechanical characteristics. The authors discovered that peak concentric and landing force and impulse between the two accelerometer placements were not significantly different, while the accelerometer placed on the hip tended to display lower vertical jump height values than the one placed on the abdomen [24].

With the vertical jump being one of the most commonly used methods for the assessment of an athlete’s lower-body muscular power and innovative accelerometer technology being an affordable and user-friendly testing modality, it is of critical importance to understand how they can be simultaneously used to adequately monitor an athlete’s performance. Thus, the purpose of the present study was to examine the impact of the anatomical accelerometer placement on the biomechanical characteristics of a countermovement vertical jump with and without an arm swing when compared to the force plate as a criterion measure.

## 2. Materials and Methods

### 2.1. Participants

Ten males ($\bar{x}$ ± SD; age = 23.5 ± 3.4 years, height = 184.4 ± 9.9 cm, body mass = 94.7 ± 16.7 kg) and seven females (age = 23.4 ± 4.5 years, height = 168.0 ± 8.6 cm, body mass = 67.1 ± 10.3 kg) volunteered to participate in this study. All participants were healthy, recreationally active individuals without current and/or previous musculoskeletal injuries that could possibly impair jumping performance. All testing procedures were approved by the University’s Institutional Review Board and all participants signed an informed consent form.

### 2.2. Testing Protocol

Upon arrival at the laboratory, each participant performed a standardized warm-up procedure consisting of a set of dynamic stretching exercises (e.g., high knees, butt kicks, forward lunges, lateral skips, and A-skips). Following the completion of the warm-up, four identical accelerometers (StriveTech, Bothell, WA, USA) sampling at 100 Hz were placed directly on the skin at the following anatomical locations: upper-back (UB; 5 cm below the C7 vertebrae), chest (CH; mid-sternum), abdomen (AB; 5 cm inferior to the umbilicus), and hip (HP; 5 cm above the greater trochanter). A disposable razor was used to remove hair from all anatomical spots where needed, and double-sided tape was used to secure accelerometers to prevent any unnecessary movement that could impact the accuracy of the data collected. Additionally, the AB and HP accelerometers were secured with elastic athletic bands and the rest with elastic bands provided by the manufacturer. To fit a diverse group of participants, the bands were offered in different sizes (i.e., small to extra-large). While standing on a uni-axial force plate system (VALD, Force Decks Max, Brisbane, Australia) sampling at 1000 Hz, each participant completed three non-sequential maximal countermovement vertical jumps with an arm swing (i.e., start with arms placed in the front of the body and elbows bent at a 90-degree angle) and three without an arm swing (i.e., hands on the hips). All four accelerometers recorded the data simultaneously with a force plate. The initiation of the vertical jump was defined as the time point at which the derived ground reaction force decreased by >20 N from the baseline (i.e., system mass), after which peak concentric force and airtime were easily detectable [25,26]. In order to minimize the possible influence of fatigue, each set was separated by a 1–2-minute rest, and each repetition was separated by a 30–60-second rest interval. The overall number of jumps performed across all participants was 126.

### 2.3. Variables

Ground reaction force curves were computed from raw vertical acceleration data by adding acceleration due to gravity (9.81 m·s^−2^) and multiplying the resultant value with the subject’s body mass, from which the following dependent variables were derived: peak concentric force (PCF; highest value observed during the concentric phase of the jumping motion), peak landing force (PLF; highest value observed during the landing phase of the jumping motion), and vertical jump height (VJH; i.e., calculated based on the flight time using the following equation [14,16]; (t^2^·g)/8; g = 9.81 m·s^−2^, t = time in the air (sec)—determined as a change in the time between the first and last time point when the ground reaction force curve crosses zero value. The same dependent variables were obtained from the force plate system software (VALD, Force Decks Max, Brisbane, Australia). The average value obtained across three jump trials from each accelerometer and the force plate system was used for statistical analysis purposes.

### 2.4. Statistical Analysis

Descriptive statistics, means and standard deviations ($\bar{x}$ ± SD) were calculated for each dependent variable. The Shapiro–Wilk test and Q–Q plots corroborated that the assumption of normality was not violated. Independent t-tests were used to examine statistically significant differences in PCF, PLF, and VJH obtained from accelerometers placed at four different anatomical locations (AB, CH, HP, and UB) and a force plate as a criterion measure, for both countermovement vertical jumps with and without an arm swing. Cohen’s d was used to calculate the measure of effect size (ES; i.e., d = 0.2 small effect, d = 0.5 moderate effect, d = 0.8 large effect) [25]. Intra-class correlation coefficients (ICC) and Bland–Altman plots based on Giavarina et al. [27] recommendations were used to calculate and graphically represent the absolute agreement between the measurements, respectively. Statistical significance was set a priori to *p* < 0.05. All statistical analyses were performed in SPSS statistical software (Version 28.0; IBM Corp., Armonk, NY, USA).

## 3. Results

**Figure 1:**
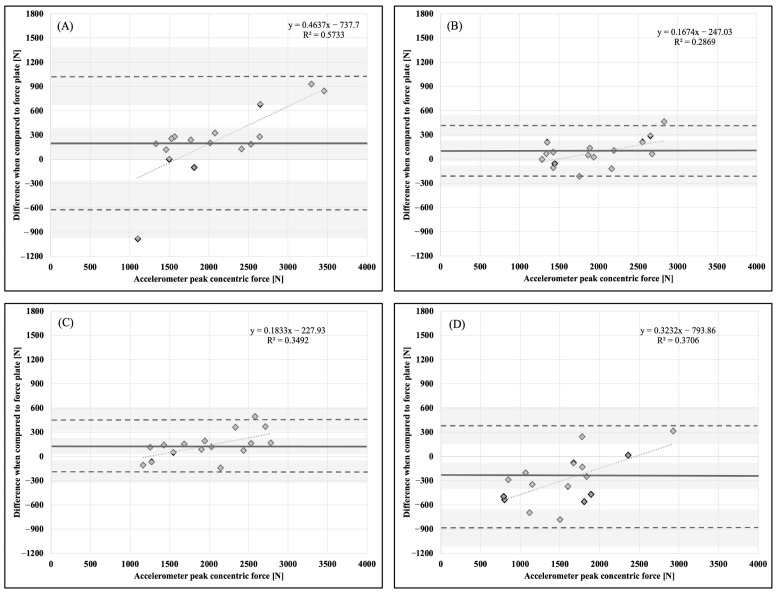
Bland–Altman plot demonstrating measurement agreement in PCF for a CVJ with no arm swing between the force plate and (**A**) abdomen, (**B**) chest, (**C**) hip, and (**D**) upper back accelerometer placement. Solid line—mean difference; dashed line—95% confidence interval; dotted line—linear regression; shaded areas—confidence interval limits for mean and agreement limits.

**Figure 2:**
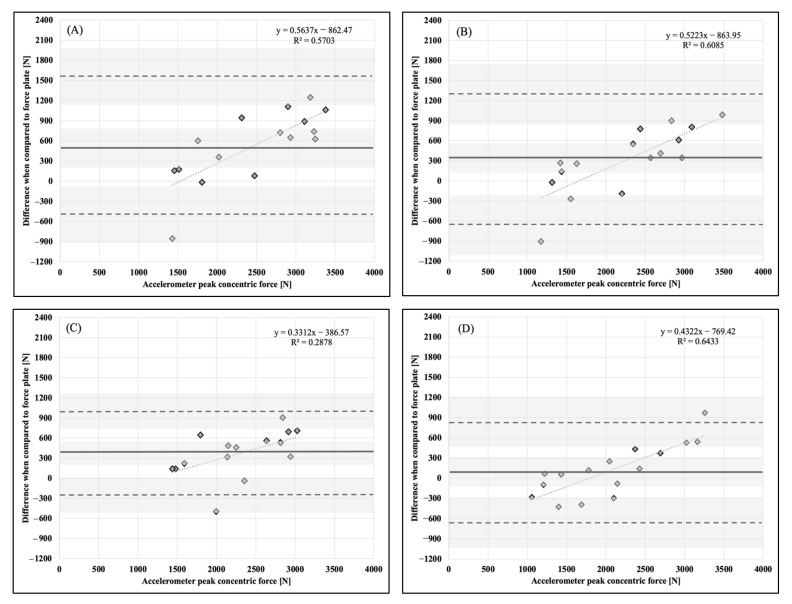
Bland–Altman plot demonstrating measurement agreement in PCF for a CVJ with arm swing between the force plate and (**A**) abdomen, (**B**) chest, (**C**) hip, and (**D**) upper back accelerometer placement. Solid line—mean difference; dashed line—95% confidence interval; dotted line—linear regression; shaded areas—confidence interval limits for mean and agreement limits.

**Figure 3:**
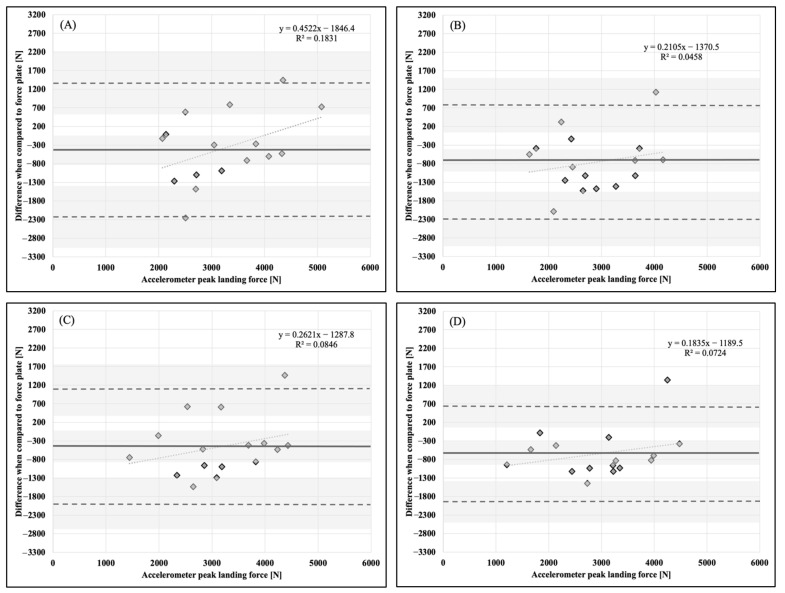
Bland–Altman plot demonstrating measurement agreement in PLF for a CVJ with no arm swing between the force plate and (**A**) abdomen, (**B**) chest, (**C**) hip, and (**D**) upper back accelerometer placement. Solid line—mean difference; dashed line—95% confidence interval; dotted line—linear regression; shaded areas—confidence interval limits for mean and agreement limits.

**Figure 4:**
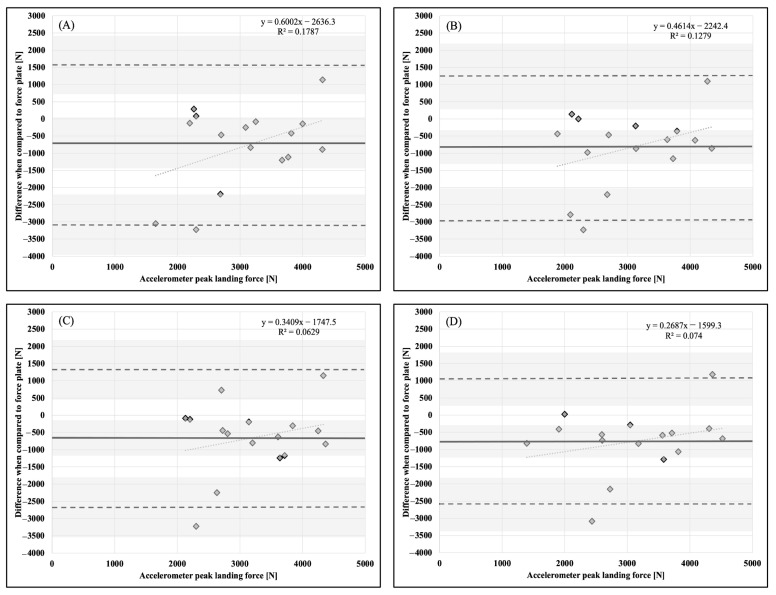
Bland–Altman plot demonstrating measurement agreement in PLF for a CVJ with arm swing between the force plate and (**A**) abdomen, (**B**) chest, (**C**) hip, and (**D**) upper back accelerometer placement. Solid line—mean difference; dashed line—95% confidence interval; dotted line—linear regression; shaded areas—confidence interval limits for mean and agreement limits.

**Figure 5:**
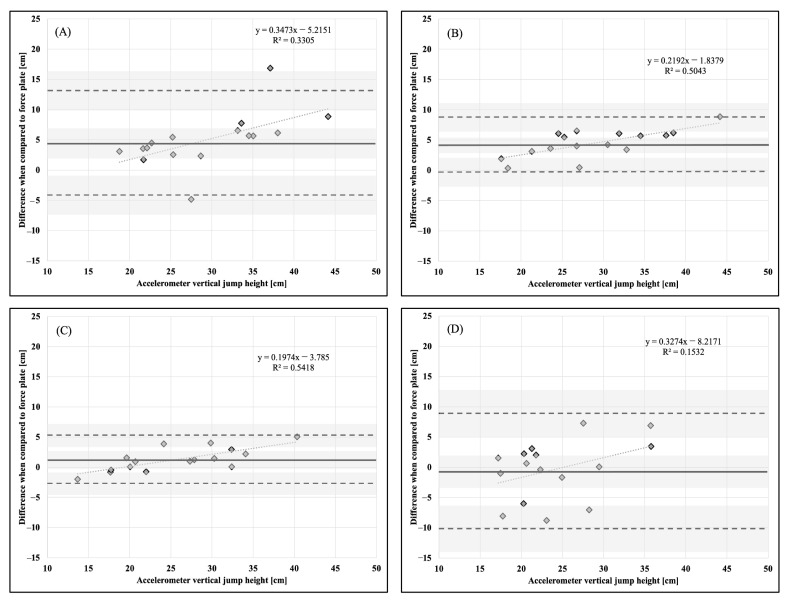
Bland–Altman plot demonstrating measurement agreement in VJH for a CVJ with no arm swing between the force plate and (**A**) abdomen, (**B**) chest, (**C**) hip, and (**D**) upper back accelerometer placement. Solid line—mean difference; dashed line—95% confidence interval; dotted line—linear regression; shaded areas—confidence interval limits for mean and agreement limits.

**Figure 6:**
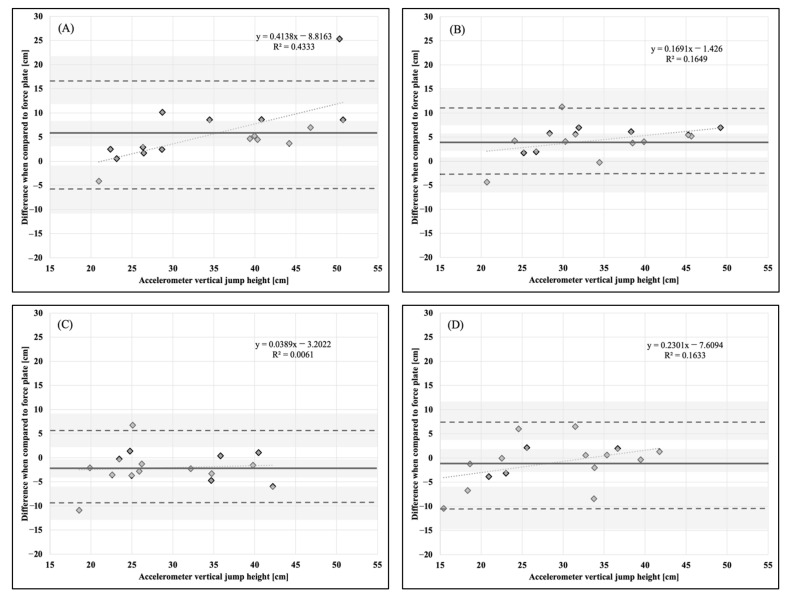
Bland–Altman plot demonstrating measurement agreement in VJH for a CVJ with arm swing between the force plate and (**A**) abdomen, (**B**) chest, (**C**) hip, and (**D**) upper back accelerometer placement. Solid line—mean difference; dashed line—95% confidence interval; dotted line—linear regression; shaded areas—confidence interval limits for mean and agreement limits.

Descriptive statistics, means, and standard deviations ($\bar{x}$ ± SD), are presented in Table 1. Bland–Altman plots for each dependent variable and accelerometer placement in comparison to the force plate as a criterion measurement are presented in Figure 1, Figure 2, Figure 3, Figure 4, Figure 5 and Figure 6.

No significant differences in PCF for countermovement vertical jumps with no arm swing were found between the force plate and AB, CH, HP, and UB. PLF was significantly lower for CH when compared to a force plate, while no differences were observed for AB, HP, and UB. Also, no significant differences in VJH were found between the force plate and CH, HP, and UB, while the AB displayed significantly greater magnitudes (Table 2).

For countermovement vertical jumps with an arm swing, a significant difference in PCF was found for AB and HP, with both being greater in magnitude when compared to the force plate as a criterion measure, while no differences were observed for CH and UB. AB, CH, and UB all displayed significantly lower PLF when compared to the force plate, while no difference was observed for HP placement. Additionally, no significant differences in VJH were observed between the force plate and AB, CH, HP, and UB (Table 3).

**Table 1:** Descriptive statistics, means and standard deviations ($\bar{x}$ ± SD), for peak concentric force (PCF), peak landing force (PLF), and vertical jump height (VJH) during countermovement vertical jumps performed with and without an arm swing.

No Arm Swing	PCF [N]	PLF [N]	VJH [cm]
Abdomen	2072.2 ± 698.4	3241.1 ± 902.2	29.3 ± 7.4 *
Chest	1923.9 ± 534.9	2851.7 ± 799.1 *	28.7 ± 7.3
Hip	1984.8 ± 547.0	3162.9 ± 875.5	25.6 ± 7.3
Upper-back	1559.0 ± 586.2	2978.8 ± 948.3	23.9 ± 5.9
Force plate	1848.9 ± 467.3	3621.8 ± 993.6	24.4 ± 6.0
**Arm Swing**	**PCF [N]**	**PLF [N]**	**VJH [cm]**
Abdomen	2471.2 ± 714.0 *	3092.1 ± 830.1 *	35.2 ± 10.1
Chest	2253.7 ± 736.7	3026.7 ± 847.3 *	33.7 ± 8.3
Hip	2323.5 ± 552.2 *	3224.0 ± 760.1	31.6 ± 8.2
Upper-back	2062.6 ± 717.3	3108.4 ± 932.6 *	28.4 ± 8.2
Force plate	1940.6 ± 468.1	3872.5 ± 1118.4	29.5 ± 7.6

Note: * significantly different when compared to force plate as a criterion measure (*p* < 0.05).

**Table 2:** Statistical analysis parameters (*p*-value; intra-class correlation coefficients—ICC; effect size—ES) for four anatomical accelerometer placements during countermovement vertical jumps without an arm swing in comparison to a force plate as a criterion measure.

PCF [N]	*p*-Value	ICC	ES
Abdomen	0.296	0.825	0.376
Chest	0.676	0.968	0.149
Hip	0.456	0.956	0.267
Upper-back	0.132	0.842	0.547
**PLF [N]**	***p*-Value**	**ICC**	**ES**
Abdomen	0.266	0.641	0.401
Chest	0.022	0.632	0.854
Hip	0.176	0.740	0.490
Upper-back	0.071	0.785	0.662
**VJH [cm]**	***p*-Value**	**ICC**	**ES**
Abdomen	0.045	0.766	0.727
Chest	0.077	0.877	0.643
Hip	0.595	0.970	0.179
Upper-back	0.865	0.803	0.084

**Table 3:** Statistical analysis parameters (*p*-value; intra-class correlation coefficients—ICC; effect size—ES) for four anatomical accelerometer placements during countermovement vertical jumps with an arm swing in comparison to a force plate as a criterion measure.

PCF [N]	*p*-Value	ICC	ES
Abdomen	0.019	0.619	0.879
Chest	0.162	0.761	0.507
Hip	0.043	0.761	0.748
Upper-back	0.573	0.883	0.201
**PLF [N]**	***p*-Value**	**ICC**	**ES**
Abdomen	0.033	0.366	1.316
Chest	0.022	0.457	1.398
Hip	0.065	0.517	1.192
Upper-back	0.044	0.648	1.149
**VJH [cm]**	***p*-Value**	**ICC**	**ES**
Abdomen	0.079	0.772	0.638
Chest	0.140	0.886	0.528
Hip	0.470	0.926	0.266
Upper-back	0.702	0.906	0.139

## 4. Discussion

To the best of our knowledge, this is the first study focused on examining the impact of the anatomical accelerometer placement on biomechanical characteristics (PCF, PLF, and VJH) during a countermovement vertical jump with and without an arm swing when compared to the force plate system as a criterion measure. The findings of the present investigation indicate that AB, CH, HP, and UB accelerometer placements displayed similar PCF values when compared to the force plate system during a countermovement vertical jump without an arm swing (i.e., hands on hips). However, CH and AB accelerometer placements tended to underestimate PLF and overestimate VJH, respectively. On the other hand, when performing a countermovement vertical jump with an arm swing, AB and HP accelerometer placements displayed greater PCF than the values obtained from the force plate system, while no differences were observed for CH and UB anatomical locations. Additionally, despite no statistically significant differences being present in VJH between different accelerometer placements and force plate system, PLF was lower in magnitude for AB, CH, and UB locations.

Previous research has reported mixed findings regarding the use of accelerometer technology for the estimation of PCF during a countermovement vertical jump with no arm swing [19,24,26]. When compared to the force plate as a criterion measure, Hojka et al. [19] found that the Myotest accelerometer placed on the hip tended to underestimate PCF on average by 167 N. In a similar investigation, Howard et al. [26] discovered that the Shimmer accelerometer placed at the same anatomical location overestimated PCF on average by 619 N. The aforementioned findings are contradictory to our results, where no statistically significant differences were observed between each of the four accelerometer placements (AB, CH, HP, and UB) and the force plate as a gold standard testing modality when performing a countermovement jump without an arm swing. However, these results seem to be in agreement with the findings of a recently conducted study that used an identical accelerometer device (StriveTech) and found similar PCF values derived from AB and HP anatomical placements (1753 N and 1776 N) [24]. The observed discrepancies may be attributed to different algorithms and sampling rates used by manufacturers of the previously mentioned accelerometer devices (e.g., 100 Hz vs. 200 Hz). On the other hand, when implementing an arm swing motion during the countermovement vertical jump, a 19–27% increase in PCF was observed for the AB and HP accelerometer placements when compared to the force plate, while CH and UB were similar in magnitude. Although further research is warranted on this topic, it is likely that wearing an accelerometer around the body region with a greater amount of soft tissue (e.g., abdomen) allowed for greater movement of the device, which ultimately resulted in the overestimation of PCF [27]. In addition, it should be noted that the upper-limb contribution to peak ground reaction force has been found to be approximately 31.5% [28]. Thus, this may provide a possible explanation for why the statistically significant differences in PCF have only been detected for AB and HP accelerometer placement when countermovement jumps were performed with an arm swing.

Another important factor to consider when examining countermovement vertical jump performance is PLF. In the present investigation, CH accelerometer placement underestimated PLF during a countermovement vertical jump with no arm swing when compared to the force plate system. However, when the arm swing motion was implemented, significantly lower PLF values were observed across all accelerometer placements (AB, CH, and UB), except for HP anatomical location. This may have occurred due to participants utilizing different landing techniques (e.g., soft landing–knee flexion > 90 deg vs. stiff landing–knee flexion < 90 deg) that have been previously defined by Devita and Skelly [29]. During softer landings, participants tend to attain greater hip and knee flexion and finish with the torso in a less erect position (e.g., lean forward) [29]. This biomechanical alteration may have allowed for greater shock absorption in contact with the ground, especially for the accelerometer placed within the upper-body region (CH and UB), ultimately resulting in underestimation of PLF during both countermovement vertical jumps with and without an arm swing [29]. Moreover, greater discrepancies observed in the arm swing condition could be attributed to the additional movement that occurred when participants added an upper-body contribution (e.g., greater PCF, PLF, and VJH). In addition, it should be noted that currently there is a considerable gap in the scientific literature focused on examining the application of accelerometer technology for quantifying PLF. In addition to the previously mentioned impact of body composition, this is another area that warrants further investigation.

This investigation also examined the differences in VJH between each accelerometer placement (AB, CH, HP, and UB) in comparison to the force plate system as a criterion measure. The AB accelerometer placement significantly overestimated VJH during countermovement vertical jump with no arm swing, while no differences were observed for the remaining anatomical locations (CH, HP, and UB). Similar observations were made by Cabarkapa et al. [16] who found that the accelerometer device placed 3 cm above the umbilicus (i.e., anterior abdomen) tended to overestimate VJH by approximately 3.1 cm during a countermovement vertical jump with no arm swing when compared to the force plate as a gold standard testing modality. The observed similarities may be attributed to researchers using the same accelerometer technology (StriveTech) for VJH assessment as well as similar anatomical placement (i.e., 5 cm vs. 3 cm inferior to the umbilicus). On the other hand, when positioned on HP, Hojka et al. [19] indicated that the Myotest accelerometer tended to overestimate VJH on average by 8 cm, while no significant differences were observed in the present investigation. This may imply that using an HP placement with the accelerometer device used in the present study may yield more accurate VJH measures than the one used by Hojka et al. [19]. In addition, when the countermovement vertical jump was performed with an arm swing, no significant differences in VJH were observed, regardless of the accelerometer anatomical location (AB, CH, HP, and UB). These findings further solidify the applicability of the accelerometer device used by the authors (StriveTech) as an accurate testing modality for the assessment of VJH.

While these findings offer additional insight into the impact of the anatomical accelerometer placement on the assessment of PCF, PLF, and VJH, this study is not without limitations. As previously indicated, body composition was not evaluated, and may have contributed to an excessive accelerometer movement that could increase the margin of measurement error. Additionally, considering that the participants examined in the present study were recreationally active individuals, future research needs to examine if our findings remain identical within a cohort of elite individual and team-sport athletes (e.g., basketball and volleyball players).

In conclusion, based on the smallest measurement error and the greatest level of agreement in comparison to a force plate as a criterion measure, the findings of the present study reveal that the most appropriate anatomical location to place the accelerometer device when attempting to estimate PCF, PLF, and VJH during a countermovement vertical jump with no arm swing are CH, AB, and UB, and during a countermovement vertical jump with an arm swing are UB, HP, and UB, respectively. Overall, these findings may help strength and conditioning professionals and sports scientists to select appropriate anatomical locations when using innovative accelerometer technologies to monitor vertical jump performance characteristics.

## Data Availability

The data presented in this study are available upon request from the corresponding authors.
